# High concentrations of environmental ammonia induced changes in large‐scale loach (*Paramisgurnus dabryanus*) immunity

**DOI:** 10.1002/ece3.7675

**Published:** 2021-05-28

**Authors:** Yun‐Long Zhang, Ze‐Hao Shang, Guang‐Yi Wang, Kun You, Di Mi

**Affiliations:** ^1^ College of Animal Science and Technology Anhui Agricultural University Hefei China

**Keywords:** air‐breathing fish, ammonia defensive, environmental ammonia loading, immunosuppression, *Paramisgurnus dabryanus*

## Abstract

High concentrations of environmental ammonia can cause reduced immunity and death in fish, causing enormous economic losses. Air‐breathing fish usually have a high ammonia tolerance and are very suitable for high‐density fish farming. However, research on the effects of environmental ammonia on air‐breathing fish immunity is lacking. Therefore, this study investigated the effects of environmental ammonia on the immunity of large‐scale loach (*Paramisgurnus dabryanus*) by exposing fish to 30 mmol/L NH_4_Cl solution and subsequently analyzing the changes in serum and liver immune indicators, including total protein, albumin, globulin, immunoglobulin (Ig) M, lysozyme, complement component (C) 3 and C4, heat shock protein (HSP) 70, HSP90, tumor necrosis factor (TNF)‐α, interleukin (IL)‐1β, IL‐6, and IL‐12. Results revealed that ammonia exposure significantly affected the total protein, albumin, globulin, IgM, complement C3 and C4, HSP70, HSP90, and inflammatory cytokine contents in the body, indicating that ammonia exposure induced a significant immune response and lowered bodily immunity. However, most of the immune indicators significantly decreased in the later stages of the experiment, suggesting a weakened immune response, which may be due to the species‐specific ammonia detoxification ability of large‐scale loach that reduces ammonia toxicity in the body.

## Graphical abstract

This study demonstrated the effects of high concentration of environmental ammonia on the immune response in *Paramisgurnus dabryanus*. Species‐specific ammonia detoxification strategies in *P. dabryanus* could weaken the immune response induced by high environmental ammonia.

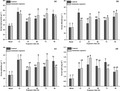

## 1 INTRODUCTION

Ammonia is a common environmental toxin found in high densities in aquaculture waters. Ammonia concentrations increase significantly as fish breeding cycles are prolonged (Chen et al., 2015). Additionally, environmental ammonia is extremely toxic to aquatic animals and causes several adverse effects (Egnew et al., 2019; Hegazi & Hasanein, 2010; Ip & Chew, 2010; Sinha et al., 2014). However, certain air‐breathing fish, such as *Paramisgurnus dabryanus* (Zhang et al., 2017, 2019), *Anabas testudineus* (Tay et al., 2006), *Bostrychus sinensis* (Ip et al., 2001; Peh et al., 2010), and *Protopterus annectens* (Loong et al., 2012), can lower the toxicity of ammonia in their environment and in the body through unique strategies of ammonia detoxification (Chew & Ip, 2014; Ip & Chew, 2010); thus, such species have an extremely high ammonia tolerance. Although air‐breathing fish adapt to relatively high concentrations of environmental ammonia, it does not mean that exposure to nonlethal concentrations of ammonia will not cause physiological damage. Due to the distinct tolerance mechanisms of ammonia exposure, the toxic effects of environmental ammonia on air‐breathing fish are often overlooked. Therefore, it is necessary to study the physiological effects of ammonia accumulation in the water on air‐breathing fish.

Large‐scale loach (*P. dabryanus*) is one of the most important economic aquaculture species in Asia (Liu et al., 2018). According to the *China Fisheries Statistics Yearbook 2020*, the production of Chinese loach in 2019 was 356,900 tons. As a typical air‐breathing fish (Zhang et al., 2016), large‐scale loach has a variety of effective strategies for ammonia detoxification (Zhang et al., 2019). Ammonia, as an environmental stressor, induces a strong stress response in large‐scale loach (Ding et al., 2019). Under stress conditions, fish adapt to the deteriorating environment through physiological changes (Jin et al., 2011). However, there is still a lack of systematic research on the physiological effects of ammonia stress on large‐scale loach. Immune response induced by environmental ammonia loading has been confirmed in various fish species and would lead to disease breakout and massive mortality (Ren et al., 2016; Zhang et al., 2018). The air‐breathing features allow large‐scale loach to have a strong hypoxic tolerance, which contributes to its high‐density breeding environment. In other words, large‐scale loach are more prone to external ammonia accumulation. Interestingly, the immune response of large‐scale loach during high level of environmental ammonia loading was different from that of non‐air‐breathing fish. Furthermore, whether the species‐specific ammonia detoxification strategies in large‐scale loach could reduce the immunosuppression induced by ammonia accumulation is a question. Therefore, this study evaluated the effects of ammonia exposure over time on the serum and liver immune indicators of large‐scale loach, aiming to clarify the effects of ammonia accumulation in the water on large‐scale loach immunity and help to the development of ammonia‐resistant species.

## 2 MATERIAL AND METHODS

### 2.1 Experimental fish

The experimental *P. dabryanus* (20.1 ± 3.2 g, 15.3 ± 2.9 cm, unsexed) were obtained from a wet market in Hefei. The fish were transferred to the laboratory and reared in five 60‐L tanks (30–50 individuals per tank) with 50 L of dechlorinated water for seven days at 23.0 ± 1.0°C before experimentation. During the rearing period, 30% of the water was changed daily, and the water was aerated by three air stones. Fish were fed with a commercial compound diet (crude protein 35%, crude lipid 7%) twice daily. Fish were left unfed during the experimental period.

### 2.2 Ammonia exposure

Fish were exposed to 30 mmol/L NH_4_Cl (i.e., calculated total ammonia 420 mg/L, calculated nonionic ammonia 3.70 mg/L) solution for various periods (6, 12, 24, 48, or 72 hr) in a volume of 10 L adjusted to pH 7.2 using NaOH solution (4 mol/L) at 25.0 ± 1.0°C. The NH_4_Cl solution was completely exchanged every 24 hr. Fish collected at the beginning of the experiment (0 hr) served as indicators of basal levels. Fish that were submerged in 10 L of dechlorinated water for various periods (12, 24, 48, 72, or 96 hr) served as controls. There were three replicated tanks with four fish in each exposure period.

### 2.3 Sampling and preparing

At the end of each exposure period (12, 24, 48, 72, or 96 hr), all the fish were anesthetized with tricaine methane sulfonate (MS‐222). Blood was quickly drawn via caudal vein puncture and then centrifuged at 4000 ɡ for 30 min at 4℃ for collecting the serum. Then, all four fish in each replicate at each exposure period were killed by a blow to the head. The fish were dissected to collect the liver. The liver samples were homogenized using a glass homogenizer placed in 4℃ ice‐cold water with 9:1 (V:W) cold normal saline solution and centrifuged at 10,000 ɡ for 20 min at 4℃ to obtain the supernatant for subsequent biochemical analyses. As the serum sample from individual fish was small, all the specimens were pooled from the four fish in each replicate and stored at −80℃ until the biochemical assay. The experiments performed on animals, the animal care, and all protocols strictly followed the guidelines of the Ethics Committee on Animal Experimentation of the Anhui Agricultural University (Hefei, China) for the care and utilization of laboratory animals.

### 2.4 Biochemical analysis

The total protein content in serum was quantified according to Robinson and Hogden (1940) using bovine serum albumin as a standard. The soluble protein content in liver samples was determined using the method reported by Bradford (1976) using bovine serum albumin as a standard. The level of serum albumin was measured via the bromocresol green method described by Webster et al. (1974). The serum globulin concentration was calculated by the following formula: globulin = total protein − albumin. Lysozyme activity was determined by a turbidimetric method (Ellis, 1990) using a commercial Assay Kit (Nanjing Jiancheng Bioengineering Institute), and the rate of change in turbidity was measured at 0.5‐ and 4.5‐min intervals at 530 nm (Kim et al., 2017).

The contents of immune globulin *M* (IgM), complement component C3 (C3), complement component C4 (C4), heat shock protein 70 (HSP70), heat shock protein 90 (HSP90), tumor necrosis factor α (TNF‐α), interleukin 1β (IL‐1β), interleukin 6 (IL‐6), and interleukin 12 (IL‐12) were determined by an enzyme‐linked immunosorbent assay (ELISA) method using a commercial assay kit (Nanjing Jiancheng Bioengineering Institute). All the test kits in the present study were specific for fish.

### 2.5 Statistic analysis

Values of the measured variables are expressed as mean ±standard error. The variance homogeneity of the data was examined using Levene's test. Data were compared by one‐way ANOVA followed by least significant difference multiple tests when significant differences were found at the 0.05 level. The *t* test was performed to compare the differences between ammonia exposure and controls at the same exposure time. Statistical analyses were performed using SPSS software (SPSS Inc.).

## 3 RESULTS

**FIGURE 1:**
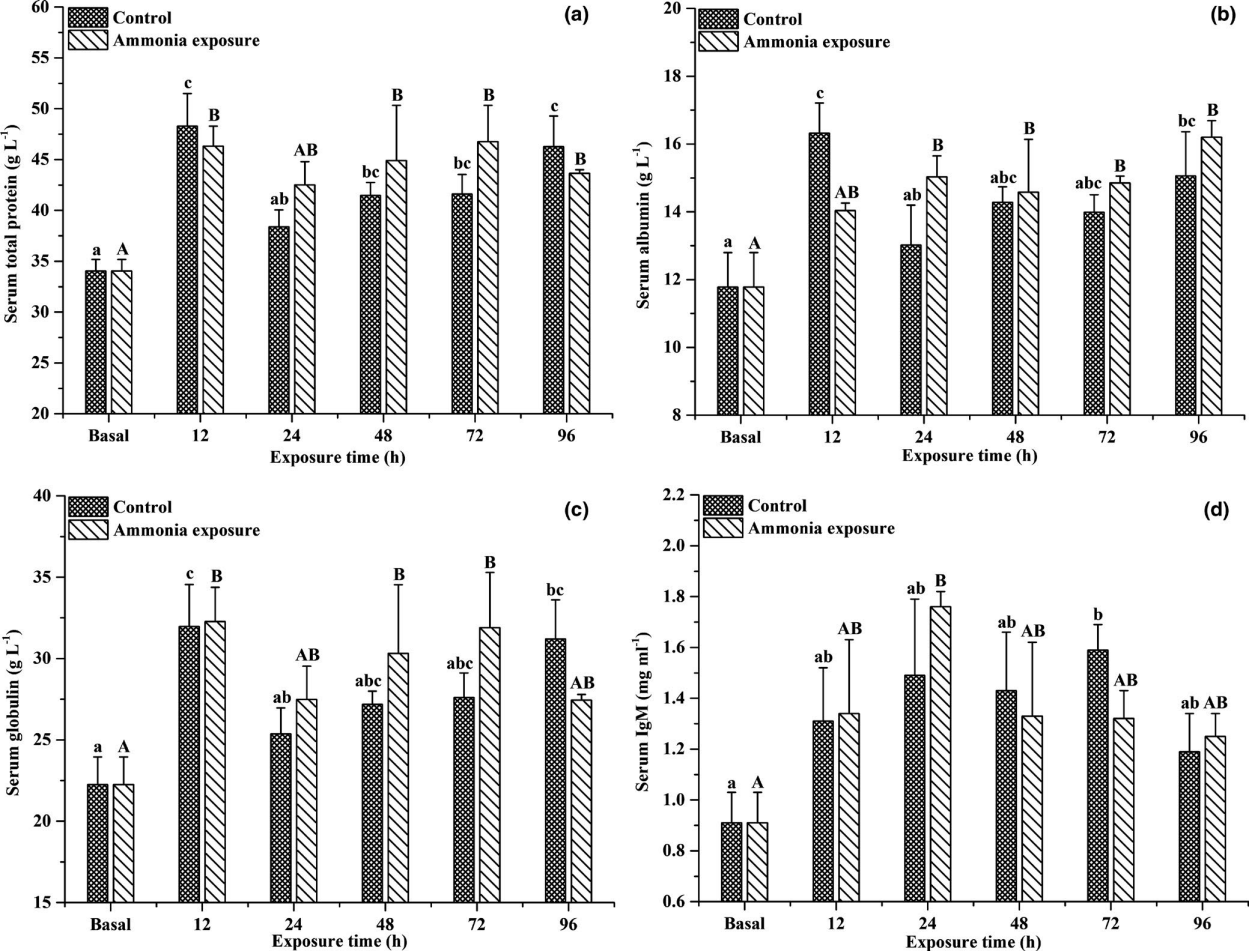
Effects of ammonia exposure on serum total protein (a), albumin (b), globulin (c), and IgM (d) contents in *Paramisgurnus dabryanus*. The different capital letters are significant differences among the different ammonia exposure times, and the different lowercase letters are significant differences among the different exposure times in the control group

Ammonia exposure over time significantly affected the serum total protein, albumin, globulin, and IgM contents of large‐scale loach (*p* < .05) (Figure 1). After exposure to 30 mmol/L NH_4_Cl solution, the serum total protein, albumin, and globulin contents of large‐scale loach increased significantly, which were significantly higher than baseline levels (*p* < .05). However, there were no significant differences detected between the ammonia exposure and control groups. After 24 hr of ammonia exposure, the serum IgM content of large‐scale loach was significantly higher than the baseline level (*p* < .05), followed by a slow decline. However, there were no significant differences detected between the ammonia exposure and control groups.

**FIGURE 2:**
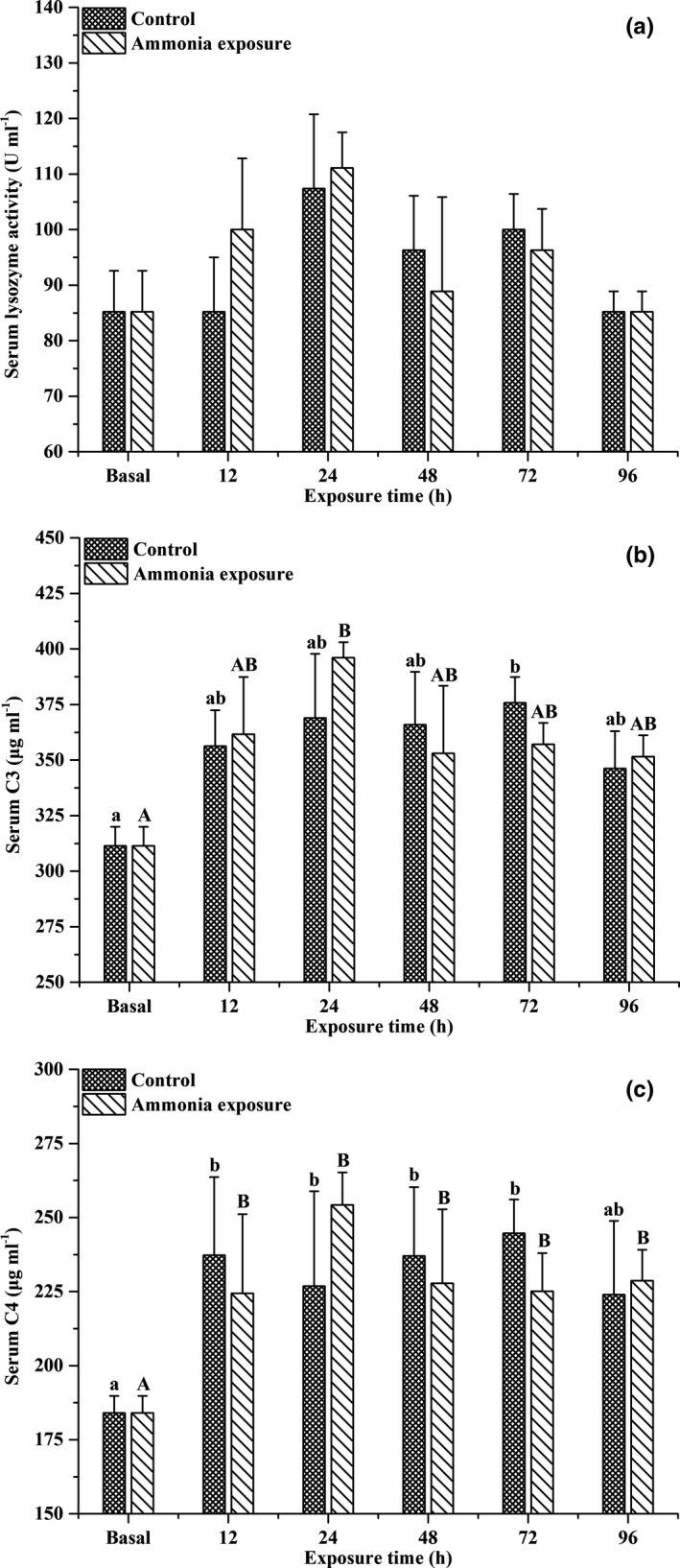
Effects of ammonia exposure on serum total lysozyme (a) activity, C3 (b), and C4 (c) contents in *Paramisgurnus dabryanus*. The different capital letters are significant differences among the different ammonia exposure times, and the different lowercase letters are significant differences among the different exposure times in the control group

**FIGURE 3:**
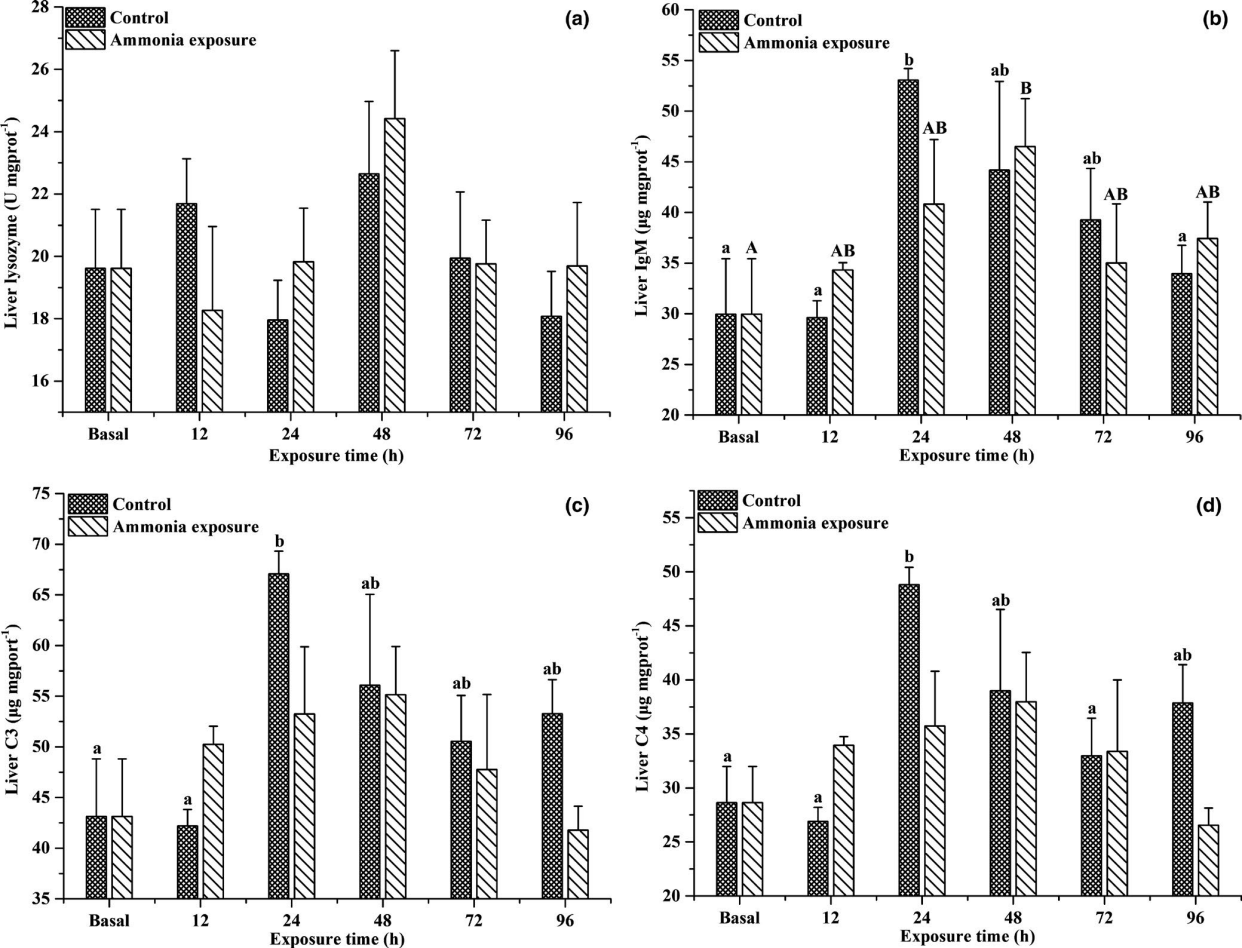
Changes in hepatic lysozyme (a) activity, IgM (b), C3 (c), and C4 (d) contents in *Paramisgurnus dabryanus* during various periods of ammonia exposure. The different capital letters are significant differences among the different ammonia exposure times, and the different lowercase letters are significant differences among the different exposure times in the control group

The effects of ammonia exposure over time on the serum lysozyme activities and complement C3 and C4 contents of large‐scale loach are shown in Figure 2. Serum lysozyme activities were not affected by ammonia exposure. As ammonia exposure prolonged, the serum complement C3 content exhibited an increasing trend at first, then decreased, and was significantly higher than the baseline level after 24 hr of ammonia exposure (*p* < .05). Ammonia exposure significantly increased the serum complement C4 content (*p* < .05), but there were no significant differences detected between the ammonia exposure and control groups. After ammonia exposure, the lysozyme activities and complement C3 and C4 contents in the liver of large‐scale loach exhibited increasing trends, then decreased (Figure 3). Exposure to 30 mmol/L NH_4_Cl solution for 48 hr caused a significant increase in IgM contents in the liver of large‐scale loach (*p* < .05), followed by a slow decline.

**FIGURE 4:**
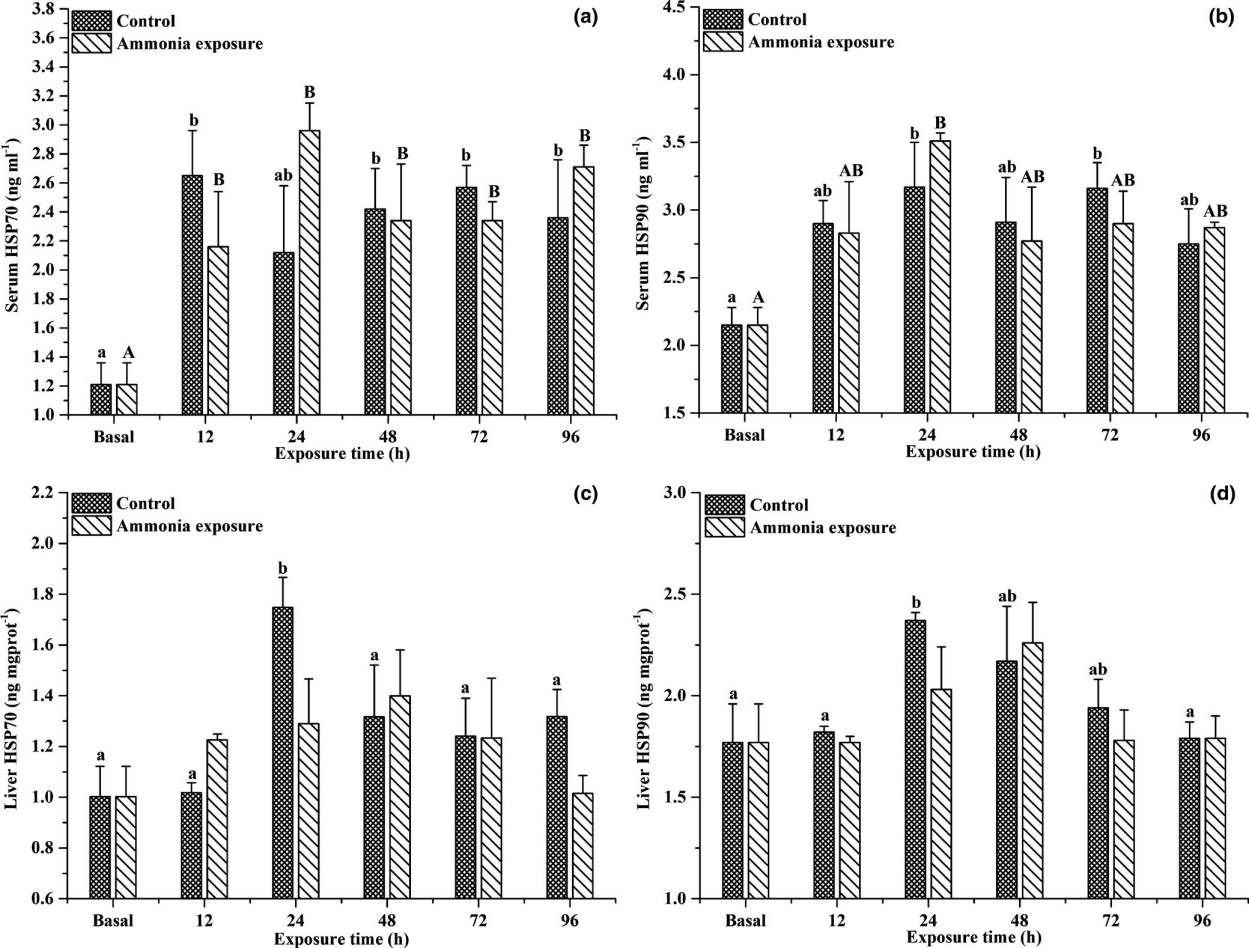
Effects of ammonia exposure on serum HSP70 (a), serum HSP90 (b), hepatic HSP70 (c), and hepatic HSP90 (d) contents in *Paramisgurnus dabryanus*. The different capital letters are significant differences among the different ammonia exposure times, and the different lowercase letters are significant differences among the different exposure times in the control group

Figure 4 shows the significant effects of ammonia exposure over time on the serum HSP70 and HSP90 levels in large‐scale loach (*p* < .05); significant effects were not detected in the liver. After ammonia exposure, the serum HSP70 and HSP90 levels exhibited an increasing trend at first, then decreased, both reaching their maximum levels within 48 hr of ammonia exposure.

**FIGURE 5:**
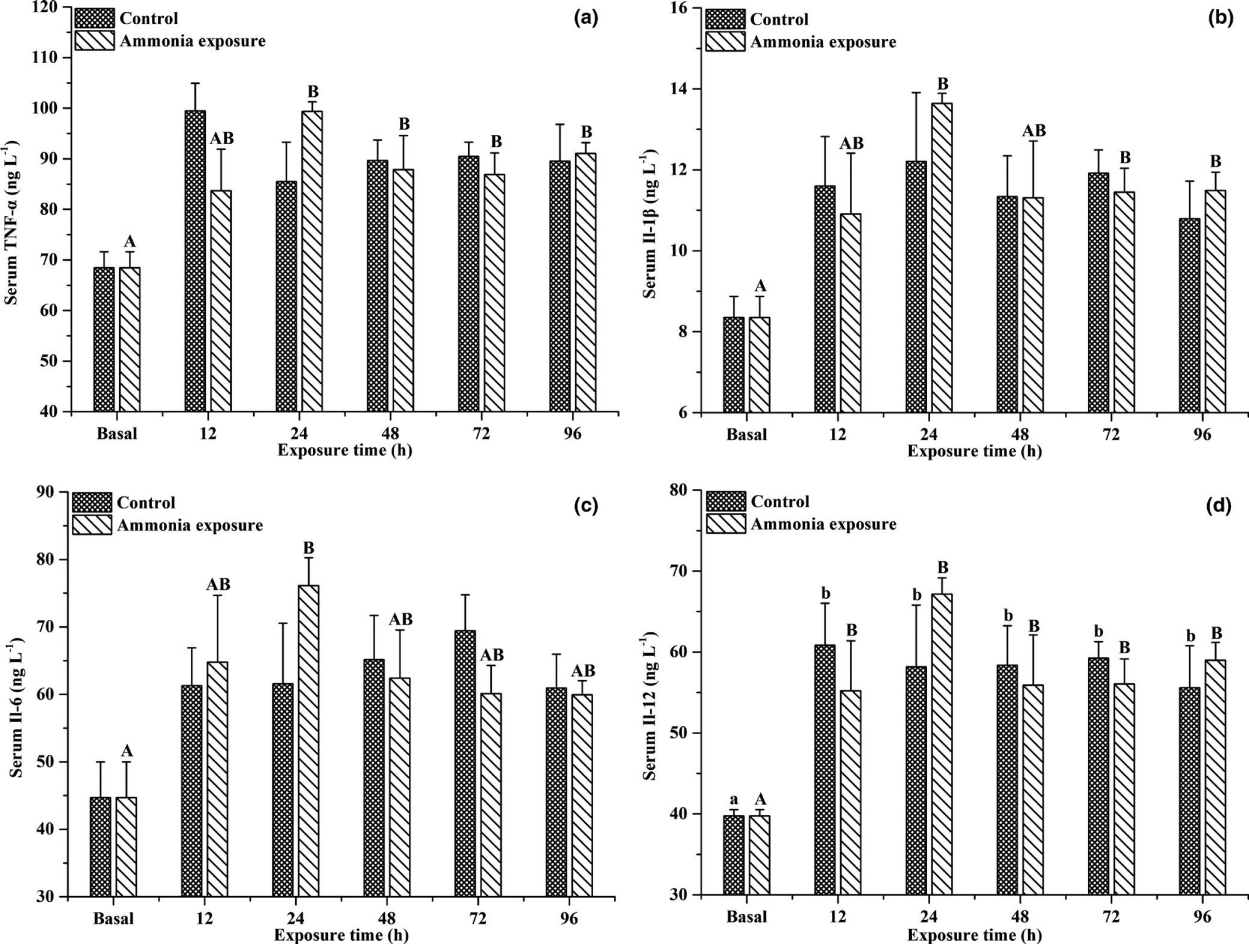
Effects of ammonia exposure on serum TNF‐α (a), IL‐1β (b), IL‐6 (c), and IL‐12 (d) contents in *Paramisgurnus dabryanus*. The different capital letters are significant differences among the different ammonia exposure times, and the different lowercase letters are significant differences among the different exposure times in the control group

**FIGURE 6:**
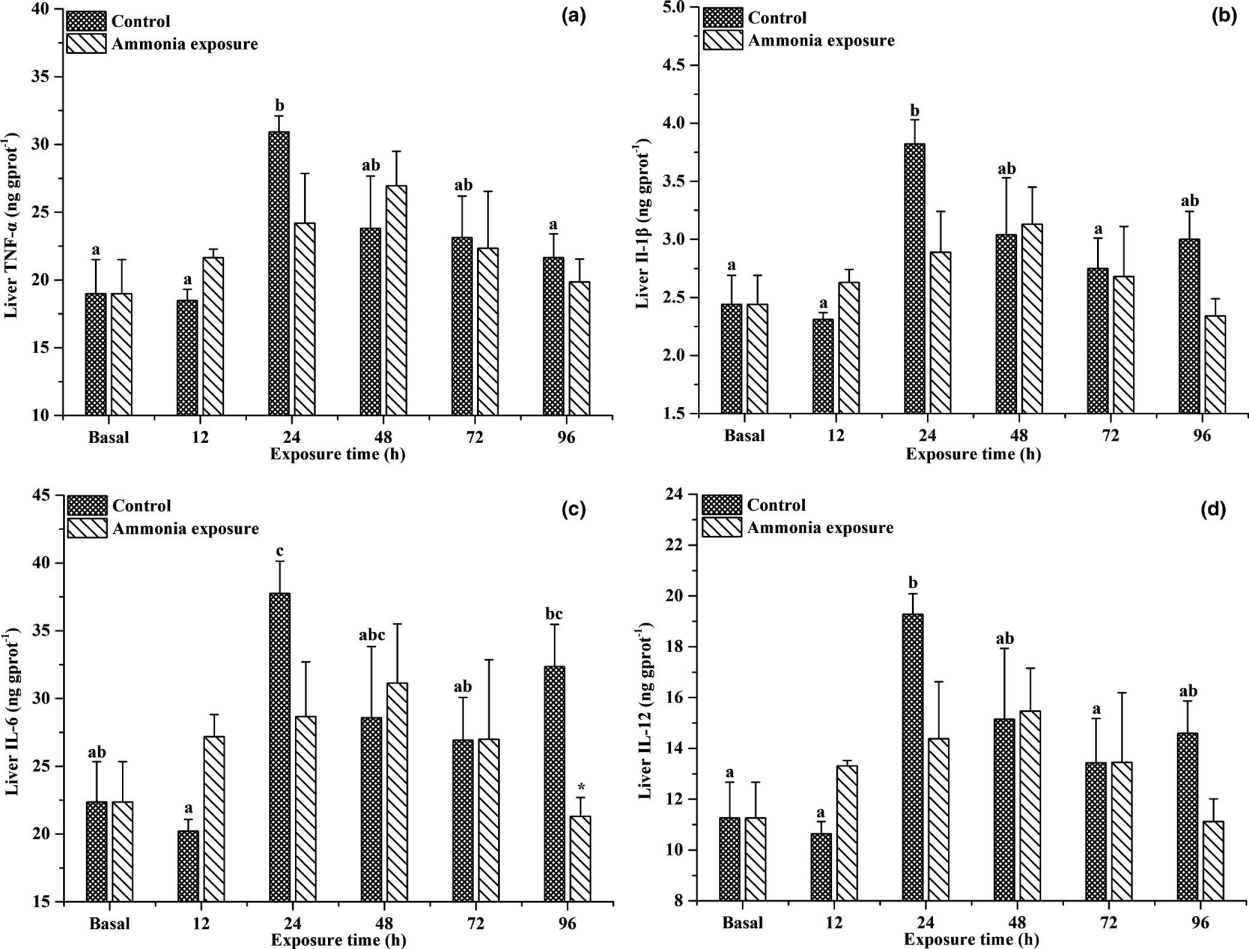
Changes in hepatic TNF‐α (a), IL‐1β (b), IL‐6 (c), and IL‐12 (d) contents in *Paramisgurnus dabryanus* during various periods of ammonia exposure. The different capital letters are significant differences among the different ammonia exposure times, the different lowercase letters are significant differences among the different exposure times in the control group; * significant difference between ammonia exposure and control groups

As shown in Figure 5, ammonia exposure significantly affected the serum levels of inflammatory cytokines, including TNF‐α, IL‐1β, IL‐6, and IL‐12 in large‐scale loach (*p* < .05). With prolonged ammonia exposure, the serum TNF‐α, IL‐1β, IL‐6, and IL‐12 levels exhibited an increasing trend at first, then decreased, all of which were significantly higher than baseline levels after 24 hr of ammonia exposure (*p* < .05). However, there were no significant differences detected between the ammonia exposure and control groups. As shown in Figure 6, after ammonia exposure, the hepatic TNF‐α, IL‐1β, IL‐6, and IL‐12 levels also exhibited slightly increasing trends, then slightly decreased; however, the levels were not significantly different from the baseline levels or control groups.

## 4 DISCUSSION

Serum biochemical factors are commonly used as indicators for evaluating fish health (Javed & Usmani, 2015; Kader et al., 2010; Ren et al., 2016; Zhang et al., 2018). In this study, ammonia exposure over time caused a significant increase in serum protein components in large‐scale loach. In *Channa punctatus*, exposure to wastewater from thermal power plants caused a significant increase in serum total protein and albumin contents, but a significant decrease in serum globulin (Javed & Usmani, 2015). Exposure to heavy metals also caused a significant increase in serum total protein and albumin contents in *Cyprinus carpio* (Gopal et al., 1997). Because the energy requirements of large‐scale loach increase significantly after ammonia exposure (Zhang et al., 2019), the increased protein contents in large‐scale loach may serve as an energy source. However, intraperitoneal injection of ammonia acetate in *Pelteobagrus fulvidraco* significantly reduced the serum total protein, albumin, and globulin contents, which was mainly due to the different experimental design where fish were fed twice a day (Zhang et al., 2018). Consequently, *P. fulvidraco* had a lower demand for endogenous energy than large‐scale loach in this study. After various periods of ammonia loading, the IgM contents of large‐scale loach significantly increased. Osteichthyes, or bonefish, also contain naturally occurring serum IgM. However, invasion by antigens in Osteichthyes induces T cells to release specific IgM (Boes, 2000; Mashoof & Criscitiello, 2016). In this study, the IgM contents of large‐scale loach increased significantly, suggesting that ammonia exposure may have induced a significant immune response.

Lysozyme is an important component of innate immunity and plays a key role in preventing bacterial invasion in fish (Saurabh & Sahoo, 2008; Zhang et al., 2020). Previous studies demonstrated that the accumulation of ammonia significantly reduced the lysozyme activities of fish, including *C. auratus* (Ren et al., 2016), *P. fulvidraco* (Zhang et al., 2018), and *Sebastes schlegelii* (Kim et al., 2015). In this study, ammonia exposure did not cause significant changes in the lysozyme activities of large‐scale loach. This result differed from a previous report that found that ammonia caused a decrease in complement C3 and C4 contents in fish (Ren et al., 2016; Zhang et al., 2018). In the present study, the increase in complement C3 and C4 contents refers to the activated complement system. In fish, ammonia accumulation in the body is largely caused by the deterioration of the water environment. Under such conditions, pathogenic microorganisms can multiply and affect fish health. Thus, activation of the complement system may be an effective strategy by which fish cope with the invasion of pathogenic microorganisms. Additionally, the effects of short‐term ammonia exposure on the activation of the fish complement system are a potential future research direction on applied immunology in fish.

Generally, the synthesis rate of HSPs significantly increases in fish that are under stress (Mohanty et al., 2018). Among them, HSP70 and HSP90 are commonly used as markers for evaluating stress (Álvarez et al., 2020; Cheng et al., 2015; Mohanty et al., 2018; Vargas‐Chacoff et al., 2018). HSP70 and HSP90 also play important roles in innate and acquired immunity in fish. The serum HSP70 and HSP90 levels in large‐scale loach significantly increased after ammonia exposure. Similar findings were observed in S. schlegelii (Kim et al., 2015), *Takifugu obscurus* (Cheng et al., 2015), and *Monopterus cuchia* (Hanqzo et al., 2017). Increasing the synthesis of HSP70 and HSP90 may be a ammonia detoxification strategy used by air‐breathing fish (Hanqzo et al., 2017). However, excessive HSPs are harmful to fish as they promote the maturation of the major histocompatibility complex, which in turn activates dendritic cells and macrophages (Quintana & Cohen, 2005; Zanin‐Zhorov et al., 2005). Thus, the reduction in ammonia toxicity in air‐breathing fish that use this strategy comes at the cost of reduced immunity. This is obviously not cost‐effective or acceptable compared with the reported ammonia tolerance strategy in large‐scale loach.

Inflammatory cytokines are also important components in the fish immune system (Vadstein et al., 2013). In this study, the serum inflammatory cytokine levels exhibited an increasing trend at first, then decreased. Similar to the results of this study, previous studies showed that the transcription levels of TNF‐α, IL‐1β, IL‐6, and IL‐12 also significantly increased in *T. obscurus* (Cheng et al., 2015) and *P. fulvidraco* (Zhang et al., 2018) after ammonia exposure. Both inflammatory responses (Collet, 2014) and environmental deterioration (Cheng et al., 2015) induce changes in inflammatory cytokines. Additionally, previous studies demonstrated that the excess production of HSPs increased TNF‐α, IL‐1β, and IL‐12 levels (Lehner et al., 2000; Mohanty et al., 2018; Panjwani et al., 2002). Collectively, the results of this study indicated that ammonia exposure induced a significant immune response in the body of large‐scale loach. However, in the later stages of the experiment, the serum levels of inflammatory cytokines significantly decreased, indicating that the immune response was significantly weakened, which may be due to the activation of species‐specific ammonia detoxification strategies in large‐scale loach.

## 5 CONCLUSION

Overall, this study demonstrated the effects of high concentration of environmental ammonia on the immune response in large‐scale loach. The total protein, albumin, globulin, IgM, complement component, HSPs, and inflammatory cytokines contents were significantly affected by various periods of high concentration ammonia exposure, suggesting that high environmental ammonia exposure induced the immunosuppression in large‐scale loach. However, in the later stages of the experiment, most of the detected immune indicators declined, indicating that the immune response was significantly weakened, which may be due to the activation of species‐specific ammonia detoxification strategies in large‐scale loach. As several feed additives (e.g., vitamin C, vitamin E, and immunopolysaccharides) can enhance immunity in fishes, further studies are suggested to focus on increasing environmental ammonia tolerance of fish induced by improving the immune system via dietary supplemental immunostimulants. Notably, pooling replicates in this study reduced the sample size and partly limited the scope of our conclusion. Further studies are suggested to sample individual fish instead of pooling replicates.

## CONFLICT OF INTEREST

All authors state that there is no conflict of interest.

## AUTHOR CONTRIBUTION

**Yun‐Long Zhang:** Conceptualization (lead); Data curation (lead); Funding acquisition (lead); Software (lead); Writing‐original draft (lead); Writing‐review & editing (lead). **Ze‐Hao Shang:** Methodology (equal). **Guang‐Yi Wang:** Methodology (equal). **Kun You:** Methodology (equal). **Di Mi:** Methodology (equal).

## Data Availability

Immune data from this study are available from the Dryad Digital Repository: https://doi.org/10.5061/dryad.0cfxpnw1r.
